# Advances in Membranes Based on PLA and Derivatives for Oil–Water Separation

**DOI:** 10.3390/polym17233135

**Published:** 2025-11-25

**Authors:** Weijun Liang, Akshay Verma, Olga Martin, Gaurav Sharma, Alberto García-Peñas

**Affiliations:** 1 Departamento de Ciencia e Ingeniería de Materiales e Ingeniería Química, IAAB, Universidad Carlos III de Madrid, Avda. de la Universidad, 30, 28911 Madrid, Spain; 2 International Research Centre of Nanotechnology for Himalayan Sustainability (IRCNHS), Shoolini University, Solan 173229, HP, India

**Keywords:** PLA, membrane, oil–water separation, preparation technique

## Abstract

The continuously growing amount of oily wastewater from industrial, domestic, and natural sources poses a major threat to water sustainability, and thus efficient oil–water separation techniques are of utmost relevance. Membrane separation has been a popular approach due to ease of handling, high performance, and versatility. Among all the membrane materials, polylactic acid (PLA) and its derivatives have been of interest as green materials because of their renewability, biocompatibility, and biodegradability. PLA possesses special merits, including low density, high permeability, and high thermal stability. Despite its advantages, PLA also has some demerits, such as brittleness, low tensile strength, and poor heat resistance. These limitations are addressed by PLA-based membranes that are generally reinforced using fillers, surface modification, and structure optimization methods. This review provides a comprehensive overview of recent developments of PLA and its derivatives for oil–water separation, with an emphasis on membrane design, fabrication methods, and porosity enhancement strategies. Some significant fabrication processes like Thermally Induced Phase Separation (TIPS), Nonsolvent-Induced Phase Separation (NIPS), and Freeze Solidification Phase Separation (FSPS) are elaborately addressed. In addition, the review emphasizes methods to improve porosity, mechanical strength, and fouling resistance while maintaining biodegradability. By reviewing recent progress and remaining challenges, this review outlines the future potential of PLA membranes and aims to inspire more research on green, efficient oil–water separation.

## 1. Introduction

Water is the fundamental basis that nurtures and sustains all life on this planet. Although it is a fundamental human need, access to clean and safe drinking water has become a key issue at the global level. Rapid industrialization, agricultural growth, and technological development spurred by population increase have drained and polluted existing freshwater resources substantially [1]. Among the numerous pollutants involved in this crisis, oil-based pollutants are of special interest. From the Industrial Revolution, the rapid growth of science, technology, and socio-economic industries has led to large-scale oil production to satisfy energy and material needs. While oil is an essential non-renewable source of energy and raw material for modern society, its extraction and use have led to serious environmental challenges. Large amounts of oily industrial waste and regular marine oil spills contaminate water bodies annually, causing serious environmental harm. Consequently, oil-based pollutants now constitute one of the primary causes of water pollution globally [2]. Industries such as textile, petrochemical, mining, and metal processing produce large amounts of oily wastewater. The pollutants usually come in various forms, including emulsified oil (<20 µm), dispersed oil (15–145 µm), and free oil (>145 µm) [3,4,5,6]. Oil spills during production and transportation also have a detrimental effect on aquatic ecosystems and cause the loss of precious natural resources. Thus, there is a need to develop energy-efficient oil–water separation technologies to treat industrial effluents and conserve water resources [7]. Several techniques have been devised for oil–water separation, which can be broadly classified into physical, chemical, and biological methods. All the methods have their advantages and limitations. Of these, membrane filtration has proven to be a successful method for oil–water separation, as it allows for the processing of high volumes of oily wastewater without the need for chemical additives. Additionally, membrane-based systems offer a more efficient and easier method than conventional separation systems [8,9]. Membrane separation processes are scalable, chemical-free, and phase-change-free, making them efficient processes ideal for portable oil–gas systems and offshore use [10]. Yet, in many cases, their operation can be limited by low water permeability and surface fouling, and they require frequent chemical cleaning and high energy input. Hence, it is highly desirable to develop highly permeable membranes with low-pressure-driven effective oil–water separation.

Recent developments in high-performance porous membrane materials have greatly broadened their applications, increasing the need for membranes with better functionality and efficiency [11]. Traditionally, most membranes are fabricated from petroleum-based polymers like PVDF [12], PP [13], PSF [14], and PES [15]. However, these materials pose environmental issues because they are not easily degradable. Although they offer several benefits, oil-based membranes are confronted with economic challenges, including high production costs and negative environmental effects, which limit their widespread industrial application. Consequently, researchers are increasingly focusing on developing renewable and environmentally friendly alternative materials [16].

Conversely, polylactic acid (PLA), a biodegradable thermoplastic polyester derived from renewable plant-based lactic acid, offers a green alternative for membrane fabrication. It offers various advantages, including low density, high surface area, good thermal insulation, high permeability, and intrinsic biodegradability and biocompatibility. It has the disadvantage of low tensile strength, brittleness, and poor resistance to heat [11]. It belongs to the aliphatic polyester class and is typically produced from hydroxy acids [17]. PLA was first synthesized in 1932 by the thermal decomposition of lactic acid under vacuum, producing a material with a relatively low molecular weight. PLA has become an extremely popular polymer due to its non-toxicity and its broad range of applications across numerous industries [18]. Additionally, it is affordable and possesses attractive properties that make it suitable for various industrial applications. Despite these merits, PLA possesses innate limitations such as brittleness and the requirement for alterations to improve its functional and structural performance [16]. In overcoming these deficits, PLA is usually blended with additives and reinforcements like natural fibers, inorganic nanoparticles, and other polymers. These modifications result in PLA-based composites that show remarkably improved mechanical strength, thermal stability, and barrier properties while preserving their biodegradability [19].

PLA membranes can be compared with other polymeric membranes used for oil–water separation, considering that these other membranes have not yet been commercialized. In this context, PLA membranes demonstrate promising results in terms of biodegradability, reducing secondary pollution commonly observed with PVDF and other polymeric membranes. They also exhibit high oil rejection efficiency, reported in some cases to reach up to 99.4%, along with high water flux and strong fouling resistance [20,21,22,23,24,25,26,27]. The use of other membranes, such as those composed of cellulose-based aerogels or graphene-based aerogels, is attracting the attention of numerous scientists due to their high porosity, high efficiency, versatility, good mechanical properties, and durability, among other advantages [28,29].

So far, several reviews have addressed different areas of polylactic acid (PLA)-derived materials, such as their preparation, drug delivery, and preparation of porous PLA membranes [11,30]. Other studies have discussed two-dimensional nanomaterial (2D-NM)-based polymeric composites for oil–water separation and methods to boost their efficiency [7]. Despite the increasing literature, there remains an inadequate number of reviews on recent developments in PLA and its derivatives for oil–water separation. The review begins with a discussion on oil–water separation membranes, followed by a summary of polymeric membrane technologies. It then delves into the production of membranes from PLA and its derivatives, emphasizing key aspects such as fiber formation techniques, surface modification methods, and approaches for enhancing porosity. The most significant preparation methods covered are Thermally Induced Phase Separation (TIPS), Nonsolvent-Induced Phase Separation (NIPS), and Freeze Solidification Phase Separation (FSPS), all of which are vital to achieving customizable membrane properties that ensure optimum oil–water separation performance. Overall, this review intends to fill the present gap by providing a comprehensive critical evaluation of recent developments in PLA-based membranes and derivatives, highlighting their potential, challenges, and future directions for developing efficient and sustainable oil–water separation technologies.

## 2. Membranes for Oil–Water Separation

**Figure 1:**
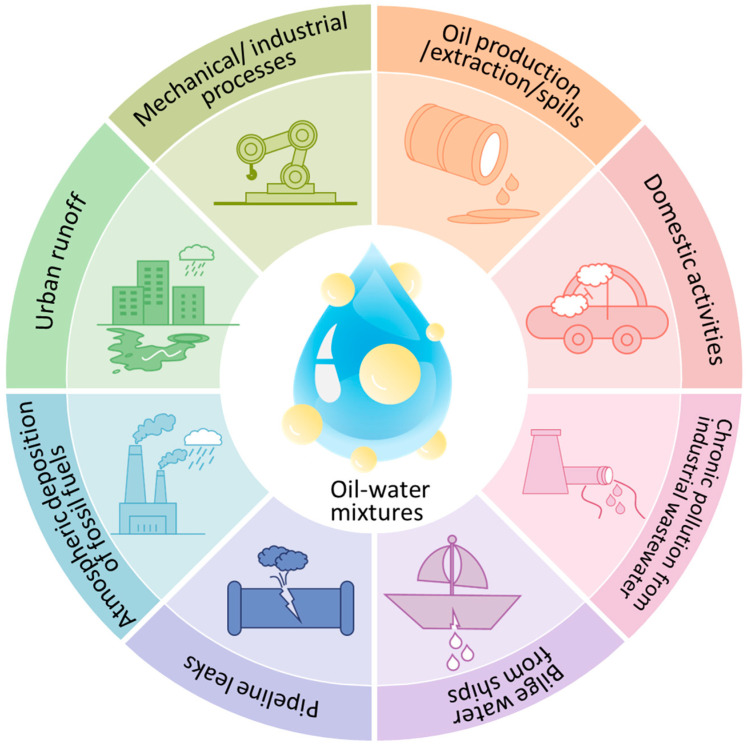
Sources responsible for oil spills.

The oil–water mixture is a common problem, not only in various mechanical and industrial processes but also in oil production and extraction, oil spills, and even domestic activities such as washing cars or equipment. As a result of these activities, between 470,000 and 8.4 million tons of oil enter the oceans each year through spills and chronic pollution, according to the Safe Drinking Water Foundation. These last two sources significantly contribute to these numbers, as numerous oil spill incidents are reported annually, along with chronic pollution from industrial wastewater, urban runoff, pipeline leaks, bilge water from ships, and atmospheric deposition of fossil fuels (Figure 1).

These mixtures form a suspension that is difficult to separate once it enters soil or natural water sources. Therefore, prevention or separation methods are necessary to avoid irreparable damage. Primarily, separating oil–water suspensions is beneficial for environmental conservation, recovering valuable resources, and improving engine efficiency. For example, irreparable damage can occur when oil–water suspensions come into contact with soil or water bodies, affecting or destroying local flora and fauna. Furthermore, recycling technology can also reclaim new raw materials for the tire and fuel industry from the oil, and the water, if recycled, can be utilized again in secondary applications like cooling or cleaning. Finally, not allowing water to touch the engine can increase its longevity, improve its performance, and prevent issues like corrosion. For this purpose, some components of the traditional processes of oil–water separation are centrifugation, flotation, settling tanks, depth filters, magnetic skimmers for oils, and magnetic separation, among others. However, these processes also have some limitations, such as process complexity, low efficiency, high energy consumption, or inefficiency in the post-treatment process. Some of the primary issues with techniques like physical precipitation, chemical flotation, and mechanical centrifugation are that they have limited practicality, and they are very expensive and rather inefficient [31]. The same goes for techniques like electrochemistry, flocculation, and gravity sedimentation in that they are also subject to secondary pollution, particularly when working with heavy oil [32].

The application of adsorption technologies has yielded extensive outcomes, especially with adsorbents from aerogels, sponges, or foams that can achieve adsorption capacities of up to 68 g/g. Many of these adsorbents are hydrophobic, showing excellent performance in oil–water separation. However, some systems, such as foams, present certain shortcomings, including low tensile strength, poor oil resistance, and the need for secondary treatment to recover the oil [32].

The use of membranes for separating oil and water holds great potential due to their high separation efficiency and relatively low cost compared to other systems [33]. Membranes also offer additional advantages, such as simple operation, low cost, and energy conservation, which contribute to environmental protection [34]. Moreover, their growing popularity in oil–water separation is also due to other functionalities such as superhydrophilicity, superoleophobicity, superhydrophobicity, and superoleophobicity, which can be modulated by the surface free energy and the micro/nano-structure of the materials [31].

Membrane systems offer multiple advantages over other technologies because they do not require thermal generation, a phase change, or active moving parts. They also combine strong anti-fouling capabilities with high separation flux [32,35]. In the past, traditional membranes lacked high selectivity due to interfacial wettability differences between oil and water, resulting in the adsorption of both components and reducing system efficiency. Nevertheless, the problems have been minimized due to better control of tunability in membrane characteristics. This means that membranes can now be specifically designed based on the nature of the oil–water mixture, allowing precise control over superhydrophobic and superoleophilic properties to achieve excellent performance [36].

## 3. Polymeric Membranes

The various advances in macromolecular chemistry have enabled the formation of membranes with distinct surface affinities and physical characteristics, best suited for oil–water separation according to specific application requirements. Table 1 outlines the materials used in oil–water separation membranes, where the significant contribution of polymers and their blends is notable. Furthermore, the table illustrates some of the benefits of these materials, which are obviously connected to their affinity and separation capacity.

Polymeric materials exhibit an interesting range of properties that can be controlled by their microstructure and composition. In this case, polymers enable control over pore size and surface area during the manufacturing process. Mechanically, polymers can offer excellent properties, including being highly flexible and possessing high durability with resistance to heat, corrosion, and adverse conditions. In most cases, these involve low costs, simplicity in preparation protocols, and high efficiency in oil–water separation processes [22,43].

One of the issues seen in polymeric materials is their limitation regarding hydrophobicity, which results in fouling and thus lowers their usability and efficiency. As a result, the permeation rate is affected, leading to increased energy consumption and a reduced lifespan. To address this, numerous research groups are working on improving surface properties by modifying various polymers and their derivatives [21].

Table 2 lists some of the main polymers used for membranes designed for oil–water separation, along with the most relevant properties that can be achieved based on the requirements dictated by the nature of the mixtures and their conditions.

Polylactic acid (PLA), also known as polylactide, is an environmentally friendly polyester synthesized from lactic acid. It is derived from renewable resources such as starch, corn, and rice through fermentation processes, and can biodegrade after use, releasing CO_2_ and water [58,62]. Compared to other matrix materials, PLA offers advantageous properties for membrane fabrication, particularly due to its ease of surface modification. When combined with good mechanical strength and processability, PLA emerges as an excellent candidate for oil–water separation membranes, as evidenced by nanofibers exhibiting high lipophilicity and strong oil adsorption capacity [22,68]. Nevertheless, there are some shortcomings associated with its limited thermal stability and lower chemical resistance, which need to be addressed through various approaches.

**Figure 2:**
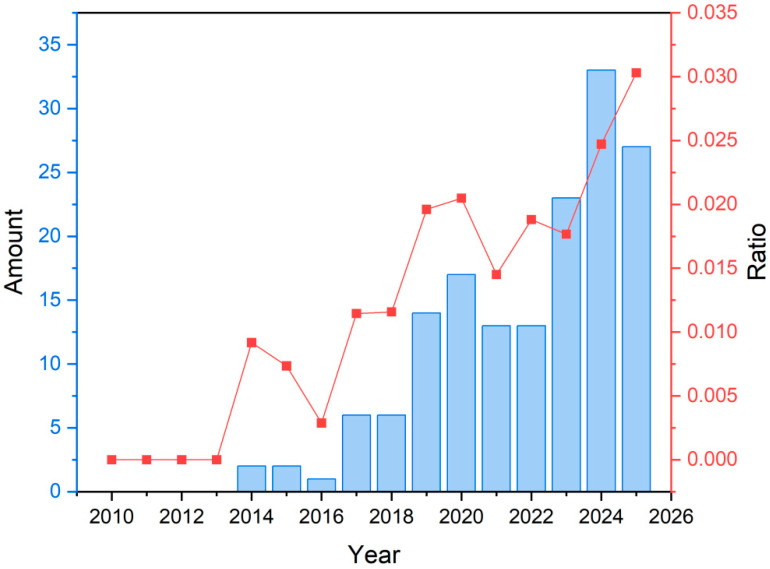
Evolution of publications obtained from the Web of Science database using “PLA & oil–water separation” as key topic terms. The ratio represents the number of publications with “PLA & oil–water separation” relative to the total number of publications with “membrane & oil–water separation”.

The importance of PLA in oil–water separation is evident, not only due to new sustainability policies promoted by the European Union, but also from a scientific perspective, where interest in the use of PLA for oil–water separation has increased exponentially since 2018 (Figure 2).

## 4. Preparation of Membranes Based on PLA and Derivatives

The methods used for preparing membranes for oil–water separation focus on controlling surface characteristics, mechanical properties, and suitability for the intended application. In this context, some of the most common preparation methods for PLA membranes for oil–water separation include electrospinning, phase separation, foaming, aerogel formation, melt blowing, and surface modification of nonwoven membranes.

### 4.1. Fiber Formation Techniques

One of the most interesting features offered by electrospinning is the ability to obtain a high surface-area-to-volume ratio, porosity, and control over the functionalization of the membranes in terms of hydrophobicity. The increase in surface area is directly related to the membrane’s adsorption capacity [69]. On the other hand, porosity and hydrophilicity are directly associated with water flux. Consequently, electrospinning represents a practical approach for fabricating membranes [70,71]. The use of PLA for membrane preparation via electrospinning, compared to other polymers, offers advantages such as tunable wettability, flexibility, and low cost. Table 3 presents some of the most recent advances in the fabrication of PLA-based membranes and derivatives through electrospinning. It includes data on surface characteristics (fiber diameter, pore size, porosity [%]), hydrophobicity (water contact angle, oil contact angle), separation performance (separation efficiency, static water contact angle under oil, oil uptake [g/g], absorption capacity [g/g], and separation flux). Yang et al. fabricated biodegradable poly(L-lactic acid) (PLLA) nanofiber membranes via electrospinning. PLA particles were dissolved in a DCM/DMF mixed solvent to form the spinning solution, which was electrospun at 25 ± 2 °C and 50 ± 5% humidity. The resulting fibers were collected on a metal drum and dried at 60 °C for 24 h. Parameters such as polymer concentration, solvent ratio, voltage, and collection distance were optimized to control fiber porosity. The optimized porous membrane was finally treated with 90% acetone for 5 min and air-dried to form the final membranes [72]. Another study by Jiang et al. prepared superoleophobic PLA/CDA composite nanofibrous membranes via electrospinning. Initially, PLA was dissolved in a CHL/DMF solvent to form spinning solutions, which were electrospun at 20 kV, a flow rate of 1 mL/h, a distance of 20 cm, and a temperature of 25 ± 3 °C. The solutions were then subjected to heat treatment at 100 °C for 2 h. Cellulose diacetate was then dissolved in acetone, and the heat-treated PLA membranes were immersed in this solution for 30 s, rinsed in deionized water for 1 h, and dried at 60 °C for 3 h to obtain the final PLA/CDA composite membranes [73].

Table 3 enables us to draw important conclusions about the various factors related to oil–water separation. As an example, pristine PLA possesses moderate oil adsorption and hydrophobicity. In such a case, the inclusion of substances like WS_2_, MoS_2_, CNTs, and CuMOF tends to be hydrophilic-reducing, characterized by water contact angles greater than 130° and increased oil adsorption. Additionally, the use of silane treatments, such as vinyltrimethoxysilane or aminopropyltrimethoxysilane, further enhances oil adsorption, with values exceeding 60 g/g. Increased surface areas can be attained by increased porosity, roughness, or, in the case of fiber, by decreasing their diameters, which normally leads to enhanced separation efficiency [69]. For instance, Table 3 illustrates fibers with diameters of 200–700 nm, which have higher separation efficiencies compared to larger fibers (>2 μm), which have lower efficiencies but better mechanical properties. The characteristics of fibers generated by electrospinning vary with the polymer solution utilized, as it determines the morphology of the fibers. In this context, viscosity plays a crucial role, affecting both fiber diameter and the degree of polymer chain entanglement.

The increase in the WCA could be attributed to the decreased diameter of fibers, which enhances the surface roughness and promotes the penetration of more air into the gaps between the fibers [76]. The wettability and absorption capacity of the membranes are improved by increasing the water contact angle. At the same time, the membranes showed excellent separation properties when they had an oleophilic feature, which is closely related to higher porosity [79]. It is lower than that of pure PLA nanofibers owing to the presence of POSS particles in the fiber surface and the pores, hence decreasing the porosity [75].

Porosity plays a crucial role and can be estimated using the Cassie–Baxter theory, which relates the differences in water contact angles between the bulk material and the electrospun surface to the presence of air pockets trapped within the structure [70,74]. The modification or functionalization of PLA surfaces with components such as POSS, LDH, carbon dots, and γ-Fe_2_O_3_ leads to higher efficiencies by enhancing flux due to changes in superhydrophobic or superoleophilic properties. The preparation of specific composites, such as PLA-γ-Fe_2_O_3_ (10 wt.%) combined with glycine, results in high water contact angles (148°), producing hydrophobic surfaces.

It is well known that flux is determined by the composition, porosity, and morphology of the membranes. Flux variations tend to increase significantly in many PLA composites by around 90%, compared to pure PLA, along with an improvement in separation efficiency. For example, the PLA-γ-Fe_2_O_3_ 40/10/17 composite exhibits a notable n-hexane flux of approximately 57,000 L/m^2^·h. PLA-based hybrids with MOFs and/or functional nanoparticles (such as WO_3_, CNTs, CQDs, etc.) can yield excellent wettability and separation efficiency/flux.

**Figure 3:**
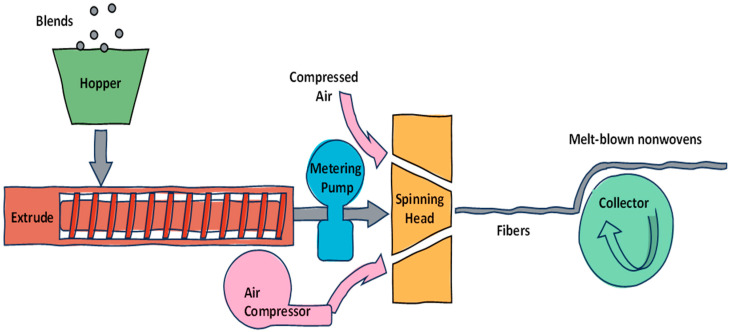
Melt-blowing process used for PLA [62].

The preparation of microfibers by blowing molten PLA is performed through nozzles using hot air. In this process, the fibers produced are coarser than those obtained by electrospinning but offer better industrial scalability. Specifically, electrospinning presents some challenges when working with thermoplastic polymers, which melt blowing can overcome. Additionally, melt blowing provides other advantages, such as high production efficiency and low pollution output [84]. Figure 3 shows the melt-blown system for PLA. The process involves melting the polymer and extruding it through a die with fine nozzles. High-velocity hot air then stretches the molten strands into microfibers, which solidify rapidly and are collected as a nonwoven mat.

Table 4 presents examples of PLA-based membranes prepared by the melt-blown process for oil–water separation. These membranes are scalable to an industrial level, solvent-free, and possess a minimal environmental footprint.

The incorporation of 10 wt.% PBAT raised the water contact angle of the membrane from 122.3° to 133.2° and made the fiber diameter distribution wider. Conversely, with 4 wt.% PBAT, the minimum fiber diameter was 2.3 μm, and the maximum adsorption capacity in oil was 5.18 g/g [62]. Upon increasing the PBE content above 10 wt.%, the melt viscosity of the polymer blend decreases, resulting in increased fluidity and a considerable reduction in fiber diameter. With increasing PBE content from 0% to 20%, the oil absorption capacity of the PLA/PBE micro-nanofiber fabric increases from 4.65 mg/g to 6.22 g/g. As the fiber diameter decreases, the volume-to-surface area ratio reduces, increasing the material’s capillary action and thus its capacity for oil absorption [85].

### 4.2. Surface Modification Techniques

**Figure 4:**
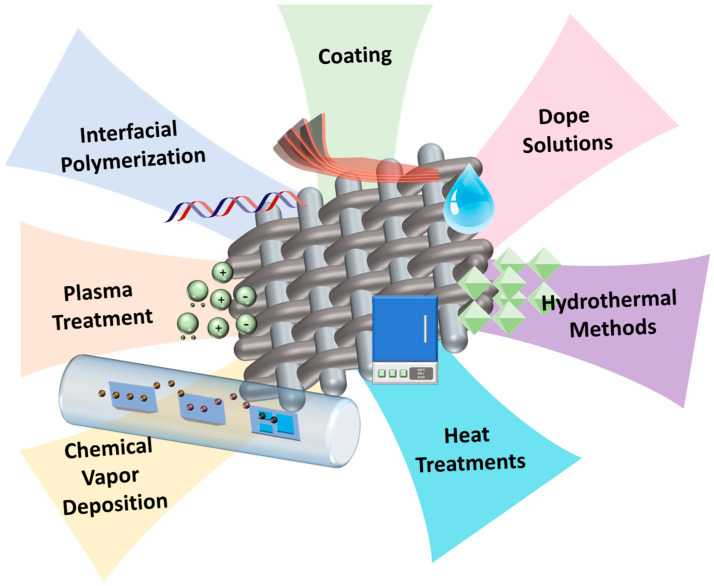
Methods for modifying membrane surfaces used for oil–water separation.

Several methods have been reported for modifying membrane surfaces for oil–water separation, including interfacial polymerization, plasma treatment, chemical vapor deposition, hydrothermal methods, dope solutions, coating, and heat treatments, among others (Figure 4) [21].

In the case of PLA, plasma treatments have shown interesting results, as PLA membranes can be modified through the introduction of functional groups. These changes promote adhesion, increase surface energy, and provide control over the membrane’s degree of hydrophobicity. Chemical vapor deposition enables PLA surface modification by depositing thin functional layers. On the other hand, surface coatings are applied to PLA membranes to impart additional properties such as photocatalysis and self-cleaning. Nevertheless, interfacial polymerization has not been explored in membranes composed of PLA. Several recent studies have utilized different surface modification techniques. For instance, Sayed et al. fabricated an environmentally friendly and biodegradable PLA@SiO_2_ nanocomposite membrane for efficient direct contact membrane distillation (DCMD). The SiO_2_ nanoparticles were derived from rice husk waste through a green synthesis route involving calcination, alkali extraction, and acid precipitation, producing fine white silica powder. A 12.5 wt.% PLA solution was electrospun into a nanofibrous membrane, while SiO_2_ nanoparticles (2 wt.%) were uniformly coated onto its surface using electrospraying to obtain the PLA@SiO_2_ membrane. To improve the structural integrity, hydrophobicity, and thermal stability, the membranes underwent post-heat treatments: heat pressing at 140 °C, annealing at 90 °C, and a combined heat press annealing process, each optimized to enhance performance and durability [86]. Another study by Scaffaro et al. developed electrospun PLA membranes and modified their surfaces using plasma treatment to enhance their performance in Streptomyces coelicolor immobilized-cell cultivations for actinorhodin production. The PLA membranes were fabricated via electrospinning and then exposed to air plasma in a radio-frequency reactor (13.56 MHz, 50 W) under controlled pressure conditions (0.5 mbar) for 30 s on each side. This plasma modification effectively altered the surface chemistry and morphology of the PLA membranes, improving their suitability for microbial adhesion and metabolite production [87].

The modification of PLA surfaces in nonwoven membranes depends on the nature of the oil–water mixture. Consequently, the surface properties of the membrane, specifically its wettability, depend on its compositional and microstructural characteristics. Higher hydrophobicity is directly related to lower surface free energy. Surface roughness also plays an important role; for example, a change in wettability can be induced by increasing surface roughness, which alters the topography. This effect is observed after a short preheating treatment in ethylene glycol, which reduces surface crystallinity [88,89]. In general, PLA nonwoven membranes exhibit good porosity and sufficient mechanical properties, enabling their use in numerous applications such as air filtration and medical protection [90,91].

Table 5 shows some of the most promising modified nonwoven membranes based on PLA for oil–water separation. It is observed that the modification of PLA with dopamine leads to hydrophilic membranes, as evidenced by the reduction in the water contact angle. The modified membrane shows a water contact angle of 58.5° compared to 121° for the pure PLA membrane. Similarly, hydrophilicity is enhanced in PLA-PDA-CNC membranes, where water absorption increases from 270% to 1000%. Moreover, the addition of CNCs into these membranes enhances the separation efficiency (99%) as well as the reusability (up to 100 cycles with an efficiency of approximately 98%) [90]. Conversely, incorporation of carbon nanotubes and AC-FAS coatings leads to the development of high water contact angles (approximately 161°). These membranes improve the separation efficiency (99.5%) as well as being reusable in a reasonable manner, with an efficiency of about 98% after 10 cycles. This is due to the phenomenal oil flux [92].

The incorporation of SiO_2_-based nanoparticles also enhances hydrophobicity, as seen in PLA-PDA-SiO_2_-PS membranes. Herein, the water contact angle is 152°, which supports huge adsorption capacities for silicone oil (32 g/g) and vegetable oil (30 g/g). These findings can be accounted for by the considerable values of flux, between 9000 and 11,500 L/m^2^·h, and good reusability [91]. Interesting findings are also seen for TiO_2_-coated membranes, which attain non-wettability even at elevated temperatures (75 °C) and exposure times of 3 to 7 s. However, the oil adsorption is constant (up to 5–7 g) with efficient reusability, maintaining high performance (10 cycles with 99% efficiency) [93].

Other methods, for example, crosslinking epoxy with PLA nanoparticles, enhance the water contact angle from 119.4° to 152.1° and deliver high separation efficiency (approximately 96%) towards oil–water mixtures. It was also observed that solvent treatments (e.g., dioxane/water) lead to super-hydrophobicity. Specifically, increasing the dioxane content raises the water contact angle from 116° to 153°, enhancing the absorption efficiency for different oils and ensuring high reusability after 10 cycles (97%) [94,95].

### 4.3. Techniques for Enhancing Porosity

There are multiple techniques to improve the porosity of PLA membranes, but foaming and aerogels are rapidly gaining attention due to their suitability for specific applications. In general, foaming is highly effective for membranes that require high flux and selective permeability. In contrast, aerogels are better suited for developing superhydrophobic properties, which are essential for achieving high oil absorption.

**Figure 5:**
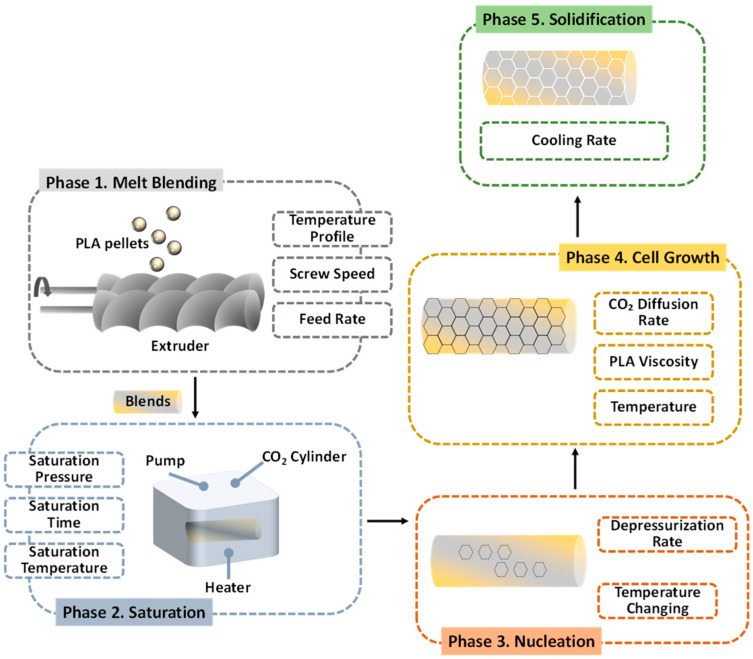
PLA foaming process.

Foaming processes are based on the introduction of gas bubbles into the polymer matrix, leading to a porous structure. For the fabrication of PLA membranes, as with many other membrane types, supercritical CO_2_ foaming is widely used. In this process, PLA is treated with CO_2_ under high pressure; when the pressure is subsequently reduced, the polymer expands, forming a uniform porous structure (Figure 5) [96]. This method is considered green (solvent-free), safe, and efficient compared to other techniques.

**Table 5:** Modified nonwoven membranes based on PLA for oil–water separation.

Membrane/Additives	Modify Method	Pore Size	Water Contact Angle * (°)	Separation Efficiency (%)	Separation Flux (L/m^2^h)	Oil Takes /Absorption Capacity	Reuseful	*Ref.*
PLA	*Immersed* in dopamine aqueous solution/CNCs/Mixture: *Hydrophobic > Hydrophilic*	223 μm	121		~1000	Water: 270%		[90]
PLA-PDA			58.5		~3000	Water: 778%		
PLA-PDA-CNCs		128 μm	0	99	3710	Water: 1000%	100 times: 98%	
PLA	*Immersed* in dopamine aqueous solution/SiO_2_ nanoparticles/PS microspheres/Mixture: *Hydrophobic > Superhydrophobic*		117					[91]
PLA-PDA			23					
PLA-PDA-SiO_2-_PS			152		Hexane: ~9000; Toluene: ~10,500 Silicone Oil: ~11,500 Pump Oil: ~7000 Vegetable Oil: ~8500	Hexane: ~25 g/g; Toluene: ~28 g/g Silicone Oil: ~32 g/g; Pump Oil: ~23 g/g Vegetable Oil: ~30 g/g; Soybean Oil: ~33 g/g	10 times: 3–6° change	
PLA	*Immersed* in TiO_2_ suspension: *Hydrophobic > Superhydrophobic*		97			ESO: ~4 g/g; Peanut Oil: ~2 g/g Xylene: ~2 g/g		[93]
PLA-TiO_2_ (55 °C/7 s)			122			ESO: ~5 g/g; Peanut Oil: ~3.5 g/g Xylene: ~2.5 g/g		
PLA-TiO_2_ (65 °C/7 s)			126			ESO: ~4.9 g/g; Peanut Oil: ~4.5 g/g Xylene: ~2.5 g/g		
PLA-TiO_2_ (75 °C/1 s)			121					
PLA-TiO_2_ (75 °C/3 s)			No Wetting					
PLA-TiO_2_ (75 °C/7 s)			No Wetting			ESO: ~4 g/g; Peanut Oil: ~3 g/g Xylene: ~2.5 g/g	10 times: >99%	
PLA-TiO_2_ (75 °C/16 s)			127					
PLA	*Immersed* in dioxane and water solvent: *Hydrophobic > Superhydrophobic*		116					[94]
PLA–dioxane–water (12 wt.%)			~135					
PLA–dioxane–water (16 wt.%)			~141°					
PLA–dioxane–water (20 wt.%)			153		N-hexane: 27; Cyclohexane: ~24 Ethanol: 17; CTC: 27		10 times: >97%	
PLA–dioxane–water (22 wt.%)			~140					
PLA	MWCNTs and AC-FAS *sprayed* on membrane: *Hydrophobic > Superhydrophobic*		140					[92]
PLA-MWCNTs (0.05 g)			~142°					
PLA-MWCNTs (0.08 g)			~150					
PLA-MWCNTs (0.1 g)			~158					
PLA-MWCNTs (0.15 g)			161	99.5	N-hexane: 59,713 Peanut Oil: ~3000 Engineering Oil: ~3000		10 times: >98.5%	
PLA-MWCNTs (0.18 g)			~159					
PLA-MWCNTs (0.2 g)			~159					
PLA	The PLA nanospheres were attached to PLA nonwoven membranes with the help of the chemical crosslinking of epoxy resin (ER)	225 μm	119.4					[95]
PLA–epoxy			133.2					
PLA–epoxy–PLA nanoparticles			140.8					
PLA nanoparticles (KH-560 treated)			152.1	96				

* Test Fluid is water.

The foaming process enables the production of membranes with controlled and relatively high porosity by adjusting physical parameters such as pressure, temperature, and time. These parameters also allow for the modulation of pore size and volume. Liquid permeability is enhanced because foaming generates interconnected pores, forming a three-dimensional (3D) network structure that significantly improves oil–water separation [97]. However, the presence of interconnected channels can sometimes lead to cell collapse, making it difficult to achieve very high porosity [98].

Table 6 presents some of the most promising foamed membranes for oil–water separation, where hydrophobicity and oleophilicity can be modulated. The highest water contact angles, directly linked to high hydrophobicity, are observed in PLA-GO membranes and PLA membranes treated with water peeling, showing values of 150.6° and 151°, respectively. In this context, PLA-GO membranes achieve a separation efficiency greater than 96%, with an oil adsorption capacity of 19.21 g/g. Even higher capacities are observed in PLA-PBS membranes (20 wt.%), reaching up to 20 g/g. Additionally, PLA membranes incorporating lignin or TPU are more easily produced while still offering competitive performance [99,100,101].

**Figure 6:**
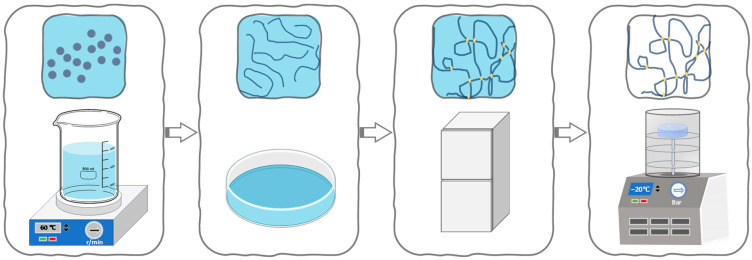
Procedure of some PLA aerogels.

Aerogels used in the preparation of PLA-based membranes enable the achievement of high porosity, large surface areas, and low densities, which enhance adsorption capacity, efficiency, selectivity, and reusability [104]. These aerogels can be prepared via supercritical drying of polymer gels or through freeze-drying. Additionally, reinforcement with silica (SiO_2_) or nanocellulose further improves mechanical strength. Figure 6 shows the preparation process of PLA aerogel. PLA is dissolved in a good solvent to form a homogeneous solution. After being cast into a membrane, the solution is cooled to a low temperature to undergo physical crosslinking, leading to gelation. The solvent is then removed via freeze-drying under vacuum to produce a PLA gel suitable for oil–water separation (Figure 6).

These aerogels confer outstanding physical properties to the membranes, including high specific surface areas and ultralow thermal conductivities. However, PLA aerogels exhibit low heat resistance, poor mechanical properties, and limited hydrolysis resistance [105]. This includes the hydrolytic degradation of the PLA aerogel, which can negatively impact the efficiency of oil–water separation and significantly reduce the service life of the PLA aerogel [104].

Table 7 presents some of the most interesting aerogels based on PLA for oil–water separation, highlighting several key facts. High-efficiency separation with high flux is reported for PLA-ZIF-8 (1 wt.%), where the separation efficiency is close to 100%, with a high contact angle (145°), and it can be used for different oils such as heptane and carbon tetrachloride. The maximum oil absorption and reusability were reported for the system based on PLLA-PDLA-PEO (50 wt.%), showing clear superhydrophobic behavior (160.7°), high porosity (around 97.9%), and an adsorption capacity of 29.9 g/g in anhydrous ethanol. On the other hand, PLA-ZIF-67 (3 wt.%) membranes present broad oil absorption across various hydrocarbons, such as petroleum ether, CCl_4_, and heptane. Finally, PLA-H_2_O + SNFs show extreme hydrophobicity for separation, with an improved porosity close to 95.6%.

### 4.4. Phase Separation Techniques

Phase separation techniques have been widely investigated in oil–water separation as a highly efficient, energy-saving and eco-friendly technical method. Phase separation can be induced by a variety of factors, including temperature [108], liquid [109], and nonsolvent [110], etc. Under these conditions, the homogeneous mixtures form continuous or discontinuous structures of different phases by nucleation and growth, which can evolve into porous or membrane materials [111]. In the application of PLA in oil–water separation, several strategies have been reported, including thermally induced phase separation (TIPS), nonsolvent-induced phase separation (NIPS) and the freeze solidification phase separation method (FSPS).

#### 4.4.1. Thermally Induced Phase Separation Method (TIPS)

TIPS exploits the thermodynamic instability of PLA solution to separate it into a polymer-rich phase and a polymer-poor phase. During the freezing process, the solvent and polymer undergo liquid–liquid (L-L) separation, reducing the homogeneity of the PLA solution into a polymer-rich phase and a solvent-rich phase. With further temperature decrease, the solvent crystallizes and separates from the polymer-rich phase (solid–liquid separation, S-L) [112]. After solvent removal, a porous membrane is formed, in which the polymer-rich phase forms the porous framework and the polymer-poor phase forms the pore structure. The outstanding advantages of TIPS include a low production cost, simple operation, and an easily tunable porous structure. Table 8 describes the PLA-based films prepared using the TIPS method for oil–water separation. Wang et al. [20] reported that the different amounts of deionized water (0, 2, 3, 4 mL) were added to the PLA/dioxane solution. After phase separation induced by thermal treatment, the addition of 4 mL of deionized water resulted in a micro/nano hybrid honeycomb-like structure, which increased the specific area from 80.34 m^2^/g to 94.4 m^2^/g. This improvement resulted in higher hydrophobicity, thereby increasing the oil absorption capacity. Li et al. [112] investigated the effects of varying the content of two PLA enantiomers (PLLA and PDLA) on oil–water separation. Increasing the PDLA fraction promoted stereo complex crystallization and facilitated a change in the pore structure. Consequently, the morphology of membranes became a complex network of submicron or smaller particles, which reduced the pore size. As the pore size decreased, the water contact angle also declined. After being frozen at −20 °C, the pore size decreased from 14.5 μm to 3.3 μm, accompanied by 9% decrease in the water contact angle and an increase in oil absorption capacity of more than 1 g/g. When the membrane is simultaneously grafted with polylactic acid (PLA) and polycaprolactone (PCL), an increase in the proportion of poly(D-lactic acid) (PDLA) leads to higher porosity and enhanced roughness, which collectively contribute to an improved oil absorption capacity [113].

#### 4.4.2. Nonsolvent-Induced Phase Separation Method (NIPS)

NIPS is a widely used technique for preparing porous polymer membranes due to its versatility, simplicity, and adaptability in shaping (Table 9) [114]. In this process, PLA is dissolved in a good solvent to obtain a homogeneous solution, which is subsequently cast into a membrane. The casting membrane is immersed in a nonsolvent bath, where solvent–nonsolvent exchange takes place, leading to a polymer-rich phase and a polymer-lean phase [115]. After drying, these two phases form a skeleton and pores, respectively, forming a continuous matrix [116]. The morphology and properties of the producing membranes are greatly influenced by several parameters, including the temperature of nonsolvent immersion and the concentration and molecular weight of the polymer, as well as the ratio and physicochemical properties of the solvent and nonsolvent system [114].

The method employs N-methyl-2-pyrrolidone (NMP) as the solvent and distilled water as the nonsolvent, where their exchange reaction induces the formation of an asymmetric, finger-like macroporous structure within the PLA membrane. Annealing treatment or the incorporation of polyhedral oligomeric silsesquioxane (POSS) effectively reduces the membrane density. From a microstructural perspective, smaller pore sizes and higher porosity are observed, which significantly improve the membrane’s performance in oil absorption capacity [117].

When n-hexane was added dropwise as a nonsolvent to a 7–17 wt.% PLA/DCM solution, the homogeneous solution underwent phase separation into a white gel. After solvent removal, porous membranes were produced by NIPS. The membrane prepared from 7 wt.% PLA exhibited the highest porosity and oil absorption capacity, approximately 6 g/g. The combination of kapok at 7 wt.% and 13 wt.% increased the oil absorption capacity, indicating that kapok enhances oil absorption. However, the effect of kapok on pore size remains unclear. This improved oil absorption capacity can be attributed to the surface wax of kapok. Subsequently, the wetting process is driven by the interaction between oil and the porous surface, resulting in a rapid initial increase in the oil absorption rate of the kapok. Over time, the oil on the kapok surface migrates and diffuses into the fiber lumen through capillary wicking [119].

Unlike most strategies for enhancing oil absorption capacity by improving membrane hydrophobicity, the addition of amino-SiO_2_-functionalized multi-walled carbon nanotubes (f-MWCNTs) to PLA results in increased hydrophilicity, as indicated by a decrease in the water contact angle from 91°for the pristine PLA membrane to 72° for the PLA/f-MWCNTs membrane. This effect can be attributed to the inherent hydrophilicity of amino-SiO_2_, which enhances its ability to attract water molecules more effectively. Simultaneously, the porosity increases from 65% to 82%, significantly improving separation efficiency and separation flux. The super-hydrophilic amino-SiO_2_ in the MWCNTs-COOH mixed-matrix solution within the membrane promotes the formation of additional nanochannels in the membrane. The presence of these additional nanochannels facilitates efficient water transport through the membrane while effectively hindering oil permeation, thereby improving the overall membrane performance [121].

#### 4.4.3. Freeze Solidification Phase Separation Method (FSPS)

The freeze solidification phase separation method involves rapidly cooling a homogeneous PLA solution to a temperature below the crystallization point of the solvent, where solvent crystallization drives phase separation, leading to the formation of voids and the organization of the polymer into a porous film with large pores, directional pore structure, lamellar structures, or dendritic channels (Table 10) [123,124]. In a composite solvent system, both the choice of solvent ratio and whether the freezing temperature is below the crystallization point of all solvent components significantly affect the pore size and the oil–water separation performance [31].

## 5. Challenges and Future Perspectives

The commercialization of PLA-based membranes is emerging within the European Union, where several suppliers, such as Nature Plast (France), Total Energies Corbion (Netherlands), and Mi-Plast Ltd. (Belgium), are actively integrating PLA into their product lines and research activities. However, specific PLA membranes designed for oil/water separation have not yet been commercialized. This is primarily due to certain technical and performance limitations, such as mechanical stability, long-term durability under wet conditions, and optimization of surface properties, which still need to be addressed before large-scale market deployment becomes feasible.

Despite polylactic acid (PLA) and its derivatives showing encouraging prospects for oil–water separation, several challenges still restrict their large-scale application and long-term stability. The primary challenge is the inherent brittleness and poor mechanical strength of PLA, which can limit the membrane’s durability and stability under continuous operation. Its inferior heat resistance and poor tolerance towards organic solvents also hinder its application in severe industrial conditions. Another persistent challenge is membrane fouling, which occurs due to the accumulation of oil droplets and organic matter on the membrane surface, leading to reduced permeability and a shorter operational lifespan. Achieving an optimal balance between hydrophilicity, mechanical strength, and biodegradability remains a significant obstacle for researchers.

The production of PLA-based membranes is also subjected to processing constraints, such as the use of organic solvents in the NIPS and TIPS methods, which are not entirely environmentally friendly and hence pose environmental and health problems. Scalability and economy remain significant barriers to commercialization, particularly when advanced modification techniques or composite fillers are employed. Most studies are limited to the laboratory scale, resulting in a lack of knowledge about membrane performance in real, complex wastewater systems.

Hence, efforts should be made in future research toward the engineering of green and energy-conserving fabrication pathways, such as solvent-free processes, melt blending, and additive manufacturing. The addition of natural or biodegradable fillers and the utilization of green surface modification processes could enhance hydrophilicity, toughness, and fouling resistance without compromising the environmental advantages of PLA. The development of innovative or responsive membranes that can adapt to varying wastewater conditions may further enhance separation performance. Moreover, stimuli-responsive and smart PLA membranes capable of responding to pH, temperature, or oil concentration variation could develop new routes for effective and selective separations. Additionally, integrating computational modeling and life cycle assessment can facilitate the rational design and the sustainability evaluation of these advanced materials.

By overcoming these issues through interdisciplinary collaboration among material scientists, chemical engineers, and environmental technologists, PLA-based membranes have the potential to become practical, sustainable, and high-performance solutions for oil–water separation, playing a crucial role in global water purification efforts.

## 6. Conclusions

Polylactic acid (PLA) and its derivatives have demonstrated significant potential as sustainable oil–water separation membranes, offering a greener alternative to the traditional petroleum-based polymers. Their natural biodegradability, renewability, and biocompatibility make them ideal for the development of environmentally friendly water treatment technologies. Current advancements in fabrication techniques such as TIPS, NIPS, and FSPS have offered better control over pore structure, surface wettability, and mechanical strength, thereby enhancing overall separation performance. However, despite these advances, PLA-based membranes remain prone to brittleness, low thermal stability, and fouling for long operations, which limit their large-scale industrial application and long-term stability. To counteract these issues, research has now begun to focus on incorporating fillers, surface functionalization, and structural optimization to achieve a balance between mechanical strength and biodegradability. In the future, the development of sustainable, solvent-free fabrication pathways and innovative surface modification methodologies will play a crucial role in driving the real-world applications of PLA-based membranes. Integrating computational modeling, life cycle assessment, and pilot-scale studies will further validate their performance and sustainability under real-world conditions. Overall, PLA-based membranes hold excellent promise toward greener, more sustainable oil–water separation technologies. With continuous innovation and interdisciplinary collaboration, these biodegradable membranes have the potential to transform wastewater treatment and make substantial contributions to global environmental protection and resource recovery.

## Figures and Tables

**Table 1:** General classification of materials used for membranes in oil–water separation [21].

Technology Term	Secondary Category	Tertiary Category	Typical Components	Advantages	*Ref.*
Membrane Materials	Inorganic Porous Materials	Ceramics	AlO_3_/ZrO_2_/TiO_2_/SiO_2_	Thermally stable Chemically resistant	[37]
		Carbon-based Materials	CNT/GO	High surface area Low density Excellent mechanical performance Chemically resistant Eco-friendly Large pore volume	[38]
		Metal–Organic Framework Membranes (MOFs)		Highly crystalline solid compounds High surface area Adjustability of the structure Unique functionality caused by flexibility Rich in physical and chemical functions Easy modifiability	[39]
	Organic Porous Materials	Polymeric Membranes	PVDF/PES/PSF/PP/PAN/ PLA/PE/PEG/PI/PTFE	High efficiency Emulsified and dispersed oil Small size Low energy requirements Cheap	[40]
		Aerogels/ Foams	Polymer-based aerogels Biomass-based aerogels Inorganic-based aerogels Carbon-based aerogels	Adjustability of the structure	[41]
	Hybrid Materials	Polymer + SiO_2_/TiO_2_ nanoparticles Polymeric Membranes + GO/MOF coating Sponge + Fe_3_O_4_/GO coating		Integrating multiple advantages	
	Natural Porous Materials	Cellulose/CA/Chitosan/Sodium alginate		Easy processing Utilization Cheap High porosity	[42]

**Table 2:** Some of the main polymers and their characteristics used for oil–water separation.

Polymer	Features	*Ref.*
Polysulfone (PSF)	High porosity, high flux and excellent mechanical properties Highly resistant to mineral acids, alkali, and electrolytes; resistant to oxidizing agents; resistant to surfactants and hydrocarbon oils; easily applicable for the conventional phase inversion processes; and able to modify the properties through blending with other polymers Hydrophobic, tendency to contaminate	[44,45,46]
Polyvinylidene difluoride (PVDF)	Hydrophilic Hydrophobic, low surface energy, outstanding chemical and thermal stability, environmentally sustainable and reusable. Forms isotropic, spherulitic microstructures that often include large macro voids	[47,48,49]
Polyether- sulfone (PES)	Hydrophobic, thermal stability and chemical resistance Small flux and poor mechanical properties less flexibility and expensive	[50,51,52]
Polypropylene (PP)	Exceptional chemical stability, filtration efficiency, and mechanical properties; large pore size Low cost, low water uptake, excellent mechanical robustness and excellent physical and chemical resistance	[53,54]
Polyimide (PI)	Excellent mechanical strength, flexibility, and stability Excellent corrosion resistance and strong separation performance	[55,56]
Polyacrylonitrile (PAN)	High hydrophilic property, rich chemical modification spots, easy obtainment of the raw material and low cost Thermal stability Good stability in solvents, excellent mechanical and film-forming properties	[57,58,59]
Polyvinyl pyrrolidone (PVP)	Hydrophilic, abundant carbonyl functional groups, nontoxic and biocompatible Facility at forming complexes, strong adhesiveness, and resistance to thermal degradation in solution	[60,61]
Polylactic acid (PLA)	Derived from the fermentation of starch, it can be obtained from cellulose, kitchen waste, or fish waste as raw materials Excellent biodegradability Eventually completely degraded into CO_2_ and H_2_O, sustainable Limited oil adsorption capacity, low adsorption selectivity, and poor mechanical properties	[31,57,62]
Cellulose	Abundance, low cost, environmental protection, biodegradability, and sustainability Improved water retention, water absorption, antibacterial, and thermal properties, nontoxic, degraded by microorganisms into natural nontoxic compounds Low flux Hydrophilicity, high specific surface area, and high mechanical strength, pore size limitation of supporting membranes, restricted the formation of a uniform structure	[63,64,65,66]
Chitosan	Low cost, environment-friendly, biodegradable, and abundant sources, special wettability, poor chemical modification ability, and harsh modification conditions	[67]

**Table 3:** Recent advances in the fabrication of PLA-based membranes via electrospinning for oil–water separation, highlighting structural characteristics (fiber diameter, pore size, porosity), surface wettability (water and oil contact angles), and separation performance (efficiency, absorption capacity, and flux).

Membrane/Additives	Fiber Diameter	Pore Size	Porosity (%)	Water Contact Angle * (°)	Oil Contact Angle (°)	Separation Efficiency (%)	Separation Flux (L/m^2^h)	Static Water Contact Angle Under Oil (°)	Oil Takes (g/g) /Absorption Capacity (g/g)	R_a_ (μm)	*Ref.*
PLA	1.2 μm		81.2	96.1		0			Motor oil: 7.75 ± 0.46	0.759 ± 0.252	[69]
PLA-MoS_2_(3 phr)	911 ± 156 nm		83.8	104.6					Motor oil: 14.66 ± 0.92	0.264 ± 0.025	
PLA-WS_2_(2 phr)	1447 ± 316 nm		86.2	108.8					Motor oil: 15.65 ± 1.24	0.351 ± 0.029	
PLA-MoS_2_-WS_2_ (1 phr/1 phr)	1649 ± 406 nm		88.2	121.4		94.68–97.17%	390–762		Motor oil: 22.45 ± 0.15	0.416 ± 0.071	
PLA	1.41 μm			110.52		>95%			Peanut oil: 31.09 Motor oil: 25.61 Silicone oil: 32.65		[71]
PLA(1 g)–Vinyltrimethoxysilane(0.1 g)	0.71 μm					>95%			Peanut oil: 60.20		
PLA(1 g)–Vinyltrimethoxysilane(0.25 g)											
PLA(1 g)–Vinyltrimethoxysilane(0.5 g)	0.62 μm								Sunflower oil: 86.90 Motor oil: 52.11 Silicone oil: 64.42		
PLA(1 g)–Aminopropyltrimethoxysilane (0.1 g)	0.22 μm			103.63				136.56 **	Peanut oil: 24.09 Motor oil: 24.15		
PLA(1 g)–Aminopropyltrimethoxysilane (0.25 g)				125.25							
PLA(1 g)–Aminopropyltrimethoxysilane (0.5 g)				104.67				162.58 **			
PLA	770 ± 330 nm								Silicon oil: 20 times deadweight		[70]
PLA–Carbon dot (3.5 w.%)	740±420 nm			Higher than PLA							
PLA–Carbon dot (21.5 w.%)	620±280 nm			Low than PLA					Silicon oil: 40 times deadweight		
PLA–Carbon dot (37.0 w.%)	720±280 nm			Low than PLA							
PLA	~700 nm		~72%	131.8							[74]
PLA-CuMOF (1.5 w.%)	~500 nm			134.5							
PLA-CuMOF (3 w.%)	~480 nm			138.1							
PLA-CuMOF (4.5 w.%)	~400 nm		~83%	140		99.47%					
PLA-CuMOF (6 w.%)	~500 nm			136.5							
PLA (8 wt./v%)	237 nm										[75]
PLA (10 wt./v%)	489 nm										
PLA (15 wt./v%)	695 nm			117.2		70%	Hexane: 351.64 ± 86.58		Palm oil: 60.45 Used engine oil: 66.03		
PLA (20 wt./v%)	1.129 μm										
PLA (15 wt./v%)- POSS (5 wt./v%)	983 nm			121.4		75%	Hexane: 338.62 ± 65.18		Palm oil: 51.10 Used engine oil: 58.78		
PLA (15 wt./v%)- POSS (5 wt./v%) coating				132.9			Hexane: 325.68 ± 56.00		Palm oil: 48.86 Used engine oil: 49.85		
PLA	6.47 μm		71.67%	122	10 ***	84.55%			Diesel oil: 11.63 Peanut oil: 9.77		[76]
PLA-CNF (2%)	~4.2 μm		73.81%			90.39%			Diesel oil: ~13 Peanut oil: ~10		
PLA-CNF (4%)	~3.7 μm		74.10%			91.63%			Diesel oil: ~13.5 Peanut oil: ~11		
PLA-CNF (6%)	~3 μm		74.47%			92.26%			Diesel oil: ~14 Peanut oil: ~11.5		
PLA-CNF (8%)	2.35 μm		73.46%			91.95%			Diesel oil: ~14.3 Peanut oil: ~12.5		
PLA-CNF (10%)	3.16 μm		76.29%	133.1		93.54%			Diesel oil: ~14.5 Peanut oil: ~13.5		
PLA-NaCl (2%)	~5.8 μm		74.46%	109.2		87.42%			Diesel oil: ~12.5 Peanut oil: ~10.4		
PLA-NaCl (4%)	~4.6 μm		76.05%			88.97%			Diesel oil: ~15 Peanut oil: ~12		
PLA-NaCl (6%)	~3.4 μm		77.70%			88.73%			Diesel oil: ~14.5 Peanut oil: ~11.7		
PLA-NaCl (8%)	~2.5 μm		72.23%			90.29%			Diesel oil: ~15.2 Peanut oil: ~11.5		
PLA-NaCl (10%)	1.69 μm		79.90%	119.2		90.14%			Diesel oil: ~15.2 Peanut oil: ~14		
PLA			86.5	133	0 ****				17.7–104.9		[77]
PLA-γ-Fe_2_O_3_ (6 wt.%)			91.2						23–268.6		
PLA-γ-Fe_2_O_3_ (8 wt.%)			92.2								
PLA-γ-Fe_2_O_3_ (10 wt.%)			92	143.1	0 ****						
PLA-γ-Fe_2_O_3_ (12 wt.%)			90.7								
PLA-γ-Fe_2_O_3_ (14 wt.%)			90.3								
PLA–γ-Fe_2_O_3_ (10 wt.%)–Glycine (0.25 wt.%)			90.3	137.7							
PLA–γ-Fe_2_O_3_ (10 wt.%)–Glycine 0.5 wt.%)			92	148							
PLA–γ-Fe_2_O_3_ (10 wt.%)–Glycine (1 wt.%)			92.4	144.4							
PLA–γ-Fe_2_O_3_ (10 wt.%)–Glycine (1.5 wt.%)			92.6	140.4							
PLA				120.7							[78]
PLA-CNT (0.25 wt.%)											
PLA-CNT (0.5 wt.%)	2.41 μm										
PLA-CNT (0.75 wt.%)	2.81 μm										
PLA-CNT (1 wt.%)	3.71 μm			133.9						1.274	
PLA (75 wt.%)-CDA (25 wt.%)	1.59 μm			0						0.864	
PLA (60 wt.%)-CDA (40 wt.%)	1.46 μm										
PLA (50 wt.%)-CDA (50 wt.%)	1.99 μm										
PLA	773 nm	1430 nm		96.96					Soybean oil: 21		[79]
PLA-CNT@LDH (1 wt.%)	764 nm	1100 nm		103.37					Soybean oil: 25		
PLA-CNT@LDH (3 wt.%)	403 nm	539 nm		109.31					Soybean oil: 28		
PLA-CNT@LDH (5 wt.%)	192 nm	414 nm		113.27					Soybean oil: 32		
PLA-ACNT@LDH (1 wt.%)	226 nm	599 nm		105.11					Soybean oil: 27		
PLA-ACNT@LDH (3 wt.%)	160 nm	361 nm		110.68					Soybean oil: 30		
PLA-ACNT@LDH (5 wt.%)	158 nm	337 nm		114.01					Soybean oil: 35		
PLA	1.125 μm			113.7	0 *****		Heptane: ~5600 Hexane: ~5000 Hexadecane: ~2600		N-heptane: 18.94 N-hexane: 19.11 Hexadecane: 18.54		[80]
PLA-WO_3_	2.946 μm			121.81	0 *****		Heptane: ~6500 Hexane: ~6000 Hexadecane: ~2800				
PLA-WO_3_-N-CQDs EDTA	8.149 μm			123.33	0 *****		Heptane: ~6600 Hexane: ~7200 Hexadecane: ~2800				
PLA-WO_3_-N-CQDs EDA	1.848 μm			132.37	0 *****		Heptane: ~11,500 Hexane: ~8200 Hexadecane: ~5800		N-heptane: 27.90 N-hexane: 35.72 Hexadecane: 32.51	25.72 nm	
PLA-γ-Fe_2_O_3_ 80/30/17	3.655 μm		90.9	135.4				151.8 ******	Castor oil: ~150; Motor oil: ~135 Silicon oil: ~133; Corn oil: ~130 Soybean oil: ~100; Olive oil: ~105 Sunflower oil: ~98		[81]
PLA-γ-Fe_2_O_3_ 70/20/17				138.06				154.24 ******	Castor oil: ~160; Motor oil: ~145 Silicon oil: ~140; Corn oil: ~125 Soybean oil: ~110; Olive oil: ~98 Sunflower oil: ~95		
PLA-γ-Fe_2_O_3_ 60/15/17				139.06				151.38 ******	Castor oil: ~200; Motor oil: ~175 Silicon oil: ~180; Corn oil: ~130 Soybean oil: ~140; Olive oil: ~105 Sunflower oil: ~110		
PLA-γ-Fe_2_O_3_ 40/10/17	2.217 μm		95.6	141.8			N-hexane: 57,324.8	156.2 ******	Castor oil:219; Motor oil: ~200 Silicon oil: ~190; Corn oil: ~145 Soybean oil: ~140; Olive oil: ~135 Sunflower oil: ~120		
PLA	312 nm		~50	~120		~98.5			Oil–water mixture: ~2400 Water-in-oil emulsion: ~320		[82]
PLA-PBAT (0.05 g)	~360 nm		~46	~125		~99			Oil–water mixture: ~290 Water-in-oil emulsion: ~490		
PLA-PBAT (0.1 g)	~415 nm		~43	~130		~99.3			Oil–water mixture: ~3100 Water-in-oil emulsion: ~500		
PLA-PBAT (0.15 g)	~450 nm		~38	~136		~99.5			Oil–water mixture: ~3800 Water-in-oil emulsion: ~520		
PLA-PBAT (0.2 g)	560 nm		~35	~155		~99.6			Oil–water mixture: ~6000 Water-in-oil emulsion: ~555		
PLA-PBAT (0.35 g)	~550 nm		~33	~145		~99.6			Oil–water mixture: ~4300 Water-in-oil emulsion: ~400		
PLA-PBAT (0.3 g)	621 nm		~33	~142		~99.3			Oil–water mixture: ~3800 Water-in-oil emulsion: ~400		
PLA			89.91	131.76		96.7	N-hexane: 1978 ± 35 CCl_4_: 1721 ± 141				[83]
PLA-PDMS/PCL (5 wt.%)			92.76								
PLA-PDMS/PCL (10 wt.%)			93.35								
PLA-PDMS/PCL (15 wt.%)			93.91	155.1	0 *******	99.3	N-hexane: 3550 ± 50 CCl_4_: 3302 ± 181				
PLA-PDMS/PCL (20 wt.%)			93.07								

* Test Fluid is water. ** Water drops in sunflower seed oil. *** Test Fluid is diesel. **** Test Fluid is diiodomethane. ***** Test Fluid is hexane. ****** Water drops in hexane. ******* Test Fluid is n-hexadecane.

**Table 4:** Membranes based on PLA prepared by the melt-blowing process for oil–water separation.

Membrane/Additives	Fiber Size	Water Contact Angle (°) *	Separation Efficiency (%)	Oil Takes /Absorption Capacity	*Ref.*
PLA	~5.5 μm	122.3	79	4.2 g/g	[62]
PLA-PBAT (2 wt.%)	~6.2 μm	125.1	82	4.5 g/g	
PLA-PBAT (4 wt.%)	~2.3 μm	128.5	88	5.18 g/g	
PLA-PBAT (6 wt.%)		130.8	91	4.9 g/g	
PLA-PBAT (8 wt.%)	~9.5 μm	132.0	94	4.7 g/g	
PLA-PBAT (10 wt.%)		133.2	96	4.5 g/g	
PLA	~5.7 μm	125		4.64 g/g	[85]
PLA-PBE (5 wt.%)	~5.7 μm	130		4.83 g/g	
PLA-PBE (10 wt.%)	~5.0 μm	134		4.69 g/g	
PLA-PBE (15 wt.%)	~3.0 μm	132		5.96 g/g	
PLA-PBE (20 wt.%)	~5.0 μm	130		6.22 g/g	

* Test Fluid is water.

**Table 6:** Some of the most promising foamed membranes for oil–water separation.

Membrane/Additives	Pore Size	Porosity (%)	Expansion Ratio	Density (g/cm^3^)	Water Contact Angle * (°)	Separation Efficiency (%)	Separation Flux (L/m^2^h)	Oil Takes/Absorption Capacity	Ref.
PLLA	28.26 μm		3.03	0.42					[98]
PLLA-8-s-PDLA-13K-3%								CCl_4_: 13.36 g/g Silicone Oil: 7.97 g/g	
PLLA-8-s-PDLA-13K-5%	20.52 μm		14.45	0.09					
PLLA-8-s-PDLA-15K-5%	27.95 μm		8.63	0.15				CCl_4_: 6.82 g/g Silicone Oil: 3.37 g/g	
PLLA-8-s-PDLA-39K-3%	23.55 μm		13.5	0.10					
PLLA-8-s-PDLA-39K-5%	14.6 μm		7.59	0.17				CCl_4_: 5.7 g/g Silicone Oil: 3.56 g/g	
PLA		~89		~0.046	~126				[102]
PLA–water (3 mL)		~90		~0.0445	~135				
PLA–water (4 mL)		90.66		0.04396	~140				
PLA–water (3 mL)/peeling					~141				
PLA–water (4 mL)/peeling					151			12–31 times its own weight	
PLA	61.7 μm		~36	~0.035	99.4			Ethyl Acetate: ~7.0 g/g	[103]
PLA–Alkaline lignin	59.7 μm		~37	~0.034	108.5			Ethyl Acetate: 4.3 g/g	
PLA–m-Alkaline lignin	73.2 μm		40.17	0.0303	130.0			Ethyl Acetate: 12.44 g/g	
PLA	197 μm		~60	69	~126			~10 g/g	[99]
PLA-PBS (3 wt.%)	177 μm		~58		~128			~12 g/g	
PLA-PBS (6 wt.%)	139.1 μm	82.1	~54	52	139			24.5 g/g	
PLA-PBS (9 wt.%)	296.8 μm		~48		~118			18.8 g/g	
PLA	115 °C: 58.2 μm 110 °C: 48.6 μm 105 °C: 17.5 μm	115 °C: 110 °C: 105 °C:	115 °C: ~58 110 °C: ~46 105 °C: ~17		113	CCl_4_: 12 (g/g)/135 min	General: 1.6–10.1 g/g		[96]
PLA-PBS (10 wt.%)	115 °C: 54.7 μm 110 °C: 57.1 μm 105 °C: 45.5 μm		115 °C: ~55 110 °C: ~57 105 °C: ~47						
PLA-PBS (20 wt.%)	115 °C: 43.6 μm 110 °C: 50.6 μm 105 °C: 47.3 μm	115 °C: 97.7	115 °C: 43.6 110 °C: ~48 105 °C: ~50		118	CCl_4_: 20 (g/g)/15 min	CCl_4_: 21.9 C_2_Cl_4_: 19.4 Ethyl Acetate:18.0 Peanut Oil: 12.7 N-Octane: 11.7 Cyclohexane:10.1 Silicone Oil: 7.9		
PLA-PBS (30 wt.%)	115 °C: 5.3 μm 110 °C: 30.7 μm 105 °C: 27.2 μm		115 °C: ~8 110 °C: ~28 105 °C: ~25						
PLA	10–50 μm				135.8				[100]
PLA-GO (1 cycle)					142.9				
PLA-GO (2 cycle)					146.2				
PLA-GO (3 cycle)					147.5				
PLA-GO (4 cycle)					150.6	>96		N-hexane: 8.11 g/g; Diethyl Ether: 8.58 g/g Cyclohexane: 9.72 g/g; Toluene: 10.41 g/g Carbon Tetrachloride: 19.21 g/g Petroleum Ether: 7.79 g/g	
PLA-GO (5 cycle)					148.2				
PLA					114.3				[101]
PLA-TPU (15 wt.%)									
PLA-TPU (30 wt.%)									
PLA-TPU (50 wt.%)					124.7			CCl_4_: 18.8 g/g; Ethyl Acetate: 11.8 g/g Silicone Oil: 8.5 g/g; Soybean Oil: 8.4 g/g cyclohexane: 6.4 g/g; gasoline: 7.7 g/g diesel: 7.2 g/g; corn oil: 4.7 g/g	

* Test Fluid is water.

**Table 7:** Aerogels involved in membranes for oil–water separation.

Membrane/Additives	Density	Pore Size	Porosity (%)	Water Contact Angle * (°)	Separation Efficiency (%)	Separation Flux (L/m^2^h)	Oil Takes /Absorption Capacity	*X*_c_ (%)	Ref.
PLA	68.53 mg/cm^3^	66.11 μm	94.0	137.6					[106]
PLA-H_2_0 (2 wt.%)-SNFs (20 wt.%)		12.63 μm		150.2					
PLA-H_2_0 (4 wt.%)-SNFs (20 wt.%)		9.72 μm		156.7					
PLA-H_2_0 (6 wt.%)-SNFs (20 wt.%)		7.78 μm		164.4					
PLA-H_2_0 (8 wt.%)-SNFs (20 wt.%)	48.08 mg/cm^3^	5.95 μm	95.58	174.6					
PLA		19.3 nm		132					[57]
PLA-ZIF-8 (0.5 wt.%)		18.6 nm		131					
PLA-ZIF-8 (1 wt.%)		17.1 nm		145	Near to 100%	Heptane–Water: 13 ** Carbon Tetrachloride–Water: 20 ** Pentane–Water: 7.1 ** N-hexane–Water: 35 **	Highest		
PLA-ZIF-8 (2 wt.%)		18.4 nm		135					
PLA-ZIF-8 (3 wt.%)		19.1 nm		133					
PLA		7.1260 nm		107					[107]
PLA-ZIF-67 (1 wt.%)		6.8410 nm		112					
PLA-ZIF-67 (2 wt.%)		8.1275 nm		117					
PLA-ZIF-67 (3 wt.%)		5.8450 nm		132	Near 100%	Petroleum Ether–Water: 71.66 *** Carb Tetrachloride–Water: 119.46 *** Heptane–Water: 59.66 *** Cyclohexane–Water: 32.56 *** Isooctane–Water: 51.18 ***	highest: 15–30 times its own weight		
PLA-ZIF-67 (4 wt.%)		7.3395 nm		121					
PLLA-PDLA		21.1 nm		140.1		Ethanol: 38.5	Anhydrous Ethanol: 19.7 g/g	44.2	[105]
PLLA-PDLA-PEO (10 wt.%)		24.2 nm	96.40%	145.4		Ethanol: 43.7		46.9	
PLLA-PDLA-PEO (20 wt.%)		22.4 nm	96.80%	149.5		Ethanol: 53.2		46.7	
PLLA-PDLA-PEO (30 wt.%)		23.1 nm	97.00%	153.2		Ethanol: 70.7		45.9	
PLLA-PDLA-PEO (40 wt.%)		27.7 nm	97.60%	156.8		Ethanol: 99.5		44.5	
PLLA-PDLA-PEO (50 wt.%)		24.2 nm	97.90%	160.7		Ethanol: 154.0	Anhydrous Ethanol: 29.9 g/g 25 times its own weight	44.6	
PLLA		52.18 μm		118.2			16.92 g/g	40.46	[104]
PLLA-PLDA (10 wt.%)		54.39 μm		124.5			18.55 g/g	33.17	
PLLA-PLDA (20 wt.%)		71.60 μm		128.3			19.1 g/g	17.45	
PLLA-PLDA (30 wt.%)		54.54 μm		131.1			20.15 g/g	8.10	
PLLA-PLDA (40 wt.%)		37.92 μm		136.7			20.53 g/g	1.08	
PLLA-PLDA (50 wt.%)		28.11 μm		140.1			20.94 g/g	0	

* Test Fluid is water. ** Oil/Water = 1:1. *** Proportion is not mentioned in the text.

**Table 8:** Thermally induced phase separation involved in membranes for oil–water separation.

Membrane/Additives	TIPS Method	Pore Size	Porosity (%)	Water Contact Angle * (°)	Oil Takes /Absorption Capacity	*X*_c_ (%)	Ref.
PLA	At −4 °C for 12 h, −80 °C for 72 h at 10 Pa	80.34 m^2^/g		~125			[20]
PLA-H_2_O (2 mL)				~130			
PLA-H_2_O (3 mL)				~132			
PLA-H_2_O (4 mL)		94.6 m^2^/g		158	Isooctane: ~15 g/g; Ethyl Acetate: ~20 g/g Acetone: ~12 g/g; Ethyl Alcohol: ~17 g/g P-xylene: ~16 g/g; Trie Thylamine: ~14 g/g Pump Oil: ~20 g/g; Engine Oil: ~15 g/g Phenixin: ~28 g/g; N-hexane: ~12 g/g Xylene: ~13.5 g/g; Cyclohexane: ~14 g/g		
PLLA/freeze	At −20 °C for 24 h	50–100 μm		111.4	~1.5 g/g	~5	[112]
PLLA-PDLA (10 wt.%)/freeze		30 μm		116.2	~1.6 g/g	~20	
PLLA-PDLA (30 wt.%)/freeze				117.7	~1.7 g/g	~45	
PLLA-PDLA (50 wt.%)/freeze				119.7	~1.8 g/g	~57	
PLLA/gelation	At 20 °C for 24 h, followed by freezing At −20 °C, followed freeze-drying	14.5 μm	66.9	98.3	~1.4 g/g	~5	
PLLA-PDLA (10 wt.%)/gelation		9.3 μm,		106.8	~1.5 g/g	~20	
PLLA-PDLA (30 wt.%)/gelation		4.0 μm		128.0	~2.4 g/g	~58	
PLLA-PDLA (50 wt.%)/gelation		3.3 μm	>80	137.2	~2.5 g/g	~62	
PDLA (50 chain lengths)-GLY	At −18 °C for 12 h, −80 °C for 24 h at 10 Pa						[113]
PDLA (70 chain lengths)-GLY-PCL		10–60 μm	94.01	145.9	13.0 g/g	21.22	
PDLA (50 chain lengths)-GLY-PCL			92.86	142.2	12.8 g/g	23.66	
PDLA (30 chain lengths)-GLY-PCL			89.14	135.5	12.1 g/g	35.9	

* Test Fluid is water.

**Table 9:** Nonsolvent-induced phase separation involved in membranes for oil–water separation.

Membrane/Additives	NIPS Method	Pore Size	Porosity (%)	Water Contact Angle * (°)	Separation Efficiency (%)	Separation Flux (LMH/bar)	Oil Takes /Absorption Capacity	*X*_c_ (%)	Ref.
PLA	Immersed in the coagulation bath filled with distilled water at ambient temperature	1.04 μm	26.43				Castor Oil: 2.96 g/g Engine Oil: ~3.0 g/g	21.16	[117]
PLA-POSS (1 wt.%)		0.82 μm	17.52				Castor Oil: 3.59 g/g Engine Oil: ~3.1 g/g	27.59	
PLA-POSS (3 wt.%)		0.41 μm	17.02				Castor Oil: ~3.56 g/g Engine Oil: 3.05 g/g	22.52	
PLA/annealing		0.81 μm	15.94				Castor Oil: 1.31 g/g Engine Oil: ~2.0 g/g	84.54	
PLA-POSS (1 wt.%)/annealing		0.17 μm	14.71				Castor Oil: ~1.85 g/g Engine Oil: ~2.35 g/g	42.12	
PLA-POSS (3 wt.%)/annealing		0.21 μm	13.37				Castor Oil: ~2.3 g/g Engine Oil: 1.81 g/g	61.69	
PLA	1. Placed on the ice, and an equal volume of C_2_H_5_OH was slowly dripped in; 2. Placed in a container containing saturated CuSO_4_ (98% relative humidity)	28.35 μm		122	73.29	N-Hexane: 7562.8 Kerosene: ~5000	N-Hexane: ~0.95 g/g Kerosene: ~1.2 g/g	38.25	[118]
PLA-Cys-CDs (1 wt.%)		24.81 μm		132		N-Hexane: ~6800 Kerosene: ~4500	N-Hexane: ~1.65 g/g Kerosene: ~2.2 g/g	39.48	
PLA-Cys-CDs (3 wt.%)		23.74 μm		140		N-Hexane: ~6300 Kerosene: ~4000	N-Hexane: ~1.55 g/g Kerosene: ~1.6 g/g	42.75	
PLA-Cys-CDs (5 wt.%)		20.33 μm		159	>99.9	N-Hexane: ~4900 Kerosene: 3157.4	N-Hexane: ~1.45 g/g Kerosene: ~1.5 g/g	47.86	
PLA-Cys-CDs (7 wt.%)		18.32 μm		151	99.98	N-Hexane: ~5200 Kerosene: ~3500	N-Hexane: ~1.2 g/g Kerosene: ~1.3 g/g	46.12	
PLA (7 wt.%)	A certain amount of n-hexane was added dropwise as nonsolvent		86.10				~6.1 g/g		[119]
PLA (10 wt.%)			76.60				~3.7 g/g		
PLA (13 wt.%)			83	128	5 μL/4 s		~4.5 g/g		
PLA (15 wt.%)			80.80				~4.1 g/g		
PLA (13 wt.%)–Kapok (0.10 wt.%)		0.09 m^3^/g					~5.5 g/g		
PLA (13 wt.%)–Kapok (0.18 wt.%)		0.077 m^3^/g		141	5 μL/0.68 s		~6 g/g		
PLA (13 wt.%)–Kapok (0.27 wt.%)		0.066 m^3^/g					~7 g/g		
PLA (7 wt.%)–Kapok (0.34 wt.%)		0.079 m^3^/g					~7.5 g/g		
PLA (7 wt.%)–Kapok (0.34 wt.%)–NaCl		0.248 m^3^/g					17.34 g/g		
PLLA-PLDA/400 μm	A coagulation bath containing deionized water			109				33.66	[120]
PLLA-PLDA/500 μm				129.5				32.78	
PLLA-PLDA/600 μm				142.9				34.26	
PLLA-PLDA/600 μm (Peeling)				151.9			Cyclohexane: ~2 s Tetrachloromethane: ~4.3 s Pump Oil: ~3.8 s; Vegetable Oil: ~3.7 s	34.26	
PLA	Submerged in a DI water coagulation bath for 24 h		65	91	94.9	Emulsion: 155 **			[121]
PLA-PVP-MWCNT			~78	85	95.1				
PLA-PVP-f-MWCNTs (0.5 wt.%)			~77	79	100	Emulsion: 281 **			
PLA-PVP-f-MWCNTs (1 wt.%)			~80	<79	98.9	Water: 485 Emulsion: 260 **			
PLA-PVP-f-MWCNTs (2 wt.%)			82	72	99.2	Water: 370			
PLA-PBAT (4 wt.%)	Immersed in water to facilitate its natural detachment from the glass surface	23 nm	50	73.71		Pure Water: 62 Oil–water: 28.6 ***			[122]
PLA–PBAT–Banana Peel (0.0125 wt.%)		25 nm	58	50.8	93				
PLA–PBAT–Banana Peel (0.025 wt.%)		~27.5 nm	61	41.3	93.3				
PLA–PBAT–Banana Peel (0.05 wt.%)		29 nm	63	38.99	95.2	Pure Water: 52.655 oil–water: 40 ***			
PLA–PBAT–Banana Peel (0.1 wt.%)		~26 nm	57	51.88	89.5	Pure Water: 32.32 oil–water: 19.8 ***			

* Test Fluid is water. ** Test Fluid is emulsion (1 g canola oil in 1 L water). *** Test Fluid is oil–water (diesel oil concentration of 100 ppm).

**Table 10:** Freeze solidification phase separation involved in membranes for oil–water separation.

Membrane/Additives	FSPS Method	Pore Size	Porosity (%)	Water Contact Angle * (°)	Separation Efficiency (%)	Separation Flux (L/m^2^h)	Oil Takes /AbsorptionCapacity	*R_a_*	Ref.
PLA	Casting	25.86 μm	51.01	117.80				23.4 nm	[31]
PLA	In low temperature (below the freezing point of DiOX)	0.46 μm	77.60	141.67	96.4	2923		78.4 nm	
PLA (H_2_0)	1. In low temperature (below the freezing point of DiOX and DCM) 2. Solution impregnation			145.73				177 nm	
PLA (acetic acid)				145.07				171 nm	
PLA (H_2_0:acetic acid = 1: 1)				151.00	99.7	16,084	n-hexane: 2.54 ± 0.55 g/g; petroleum ether: 2.93 ± 0.21 g/g ethyl acetate: 4.72 ± 0.28 g/g; methylbenzene: 5.37 ± 0.14 g/g	338 nm	
PLA (DiOX: DCM = 10:0)	In low temperature (below the freezing point of DiOX and DCM)	0.54 μm				2923			
PLA (DiOX: DCM = 9:1)		0.98 μm				6266			
PLA (DiOX: DCM = 4:1)		1.32 μm			95.1	8287			
PLA (DiOX: DCM = 1:1)		0.35 μm				4278			
PLA (DiOX: DCM = 0:10)						379			

* Test Fluid is water.

## Data Availability

No new data were created or analyzed in this study.

## Abbreviations

The following abbreviations are used in this manuscript:

PVDF	Polyvinylidene fluoride
LDH	Layered double hydroxides
CO_2_	Carbon dioxide
PP	Polypropylene
CNTs	Carbon nanotubes
PCL	Polycaprolactone
PSF	Poly sulfone
CNF	Cellulose nanofiber
PES	Polyether sulfone
WCA	Water contact angle
PLA	Polylactic acid
PBE	Propylene-based elastomer
2D-NM	2-Dimensional Nanomaterial
GO	Graphene oxide
